# Sarcopenia/Muscle Mass is not a Prognostic Factor for Short- and Long-Term Outcome After Esophagectomy for Cancer

**DOI:** 10.1007/s00268-016-3603-1

**Published:** 2016-06-08

**Authors:** Brechtje A. Grotenhuis, Joël Shapiro, Stefan van Adrichem, Marianne de Vries, Marcel Koek, Bas P. L. Wijnhoven, J. Jan B. van Lanschot

**Affiliations:** 1 Department of Surgery, Erasmus MC, University Medical Center, PO Box 2040, CA 3000 Rotterdam, The Netherlands; 2 Department of Radiology and Medical Informatics, Erasmus MC, University Medical Center, Rotterdam, The Netherlands

## Abstract

**Background:**

Recent studies have suggested that sarcopenia is a prognostic risk indicator of postoperative complications and predicts survival in cancer patients. The aim of this study is to investigate whether sarcopenia is associated with postoperative short-term outcome (morbidity and mortality) and long-term survival in patients undergoing esophagectomy for cancer after neoadjuvant chemoradiotherapy.

**Methods:**

All patients who underwent neoadjuvant chemoradiotherapy followed by esophagectomy for cancer, and of whom an adequate CT scan was available, were included in the current study. The presence of sarcopenia was defined by CT imaging using cut-off values of the total cross-sectional muscle tissue measured transversely at the third lumbar level.

**Results:**

A total number of 120 patients were eligible for analysis. Almost half of the patients (*N* = 54, 45 %) were classified as having sarcopenia; 24 sarcopenic patients (44 %) had overweight and 5 sarcopenic patients (9 %) were obese. Overall morbidity and mortality rate did not differ significantly between sarcopenic and non-sarcopenic patients, nor did long-term overall or disease-free survival. Also sarcopenic obesity was not associated with worse outcome.

**Conclusion:**

The presence of sarcopenia was not associated with a negative short- and long-term outcome in this selected group of esophageal cancer patients after neoadjuvant chemoradiotherapy followed by esophagectomy.

## Introduction

The increasing incidence of esophageal cancer is at least partly due to the rising obesity rates worldwide. Although there is a direct causality between obesity and the incidence of esophageal cancer, studies have shown that BMI is not an independent prognostic factor for short- and long-term outcomes after esophagectomy [1, 2]. More recently, the focus of preoperative risk assessment has shifted towards the concept of frailty. Frailty can be defined as a syndrome of decreased reserve and resistance to stressors, resulting from cumulative declines across multiple physiologic systems, and causing vulnerability to adverse outcomes [3]. An important feature of the frailty syndrome is loss of muscle tissue referred to as sarcopenia [4].

Sarcopenia encompasses the condition of decreased muscle mass and the loss of function due to muscle wasting. Depletion of skeletal muscle mass can occur in normal, underweight, or overweight patients, and therefore does not equal ordinary weight loss or cachexia. It has been hypothesized that sarcopenia may reflect a state of prolonged catabolism or increased metabolic activity of a more aggressive tumor biology leading to systemic inflammation causing muscle wasting and poor postoperative outcome. However, the molecular mechanisms of sarcopenia remain understood poorly. Sarcopenia is an important factor in functional compromise as it leads to less physical activity, which in turn leads to more profound sarcopenia: a vicious circle to functional decline [5–8]. Several studies have examined the relationship between cancer and sarcopenia. A recent review investigating core muscle size measured with abdominal CT scans and outcome after major abdominal surgery suggested that this assessment is an objective and robust prognostic risk indicator of postoperative complications and mortality [9]. Furthermore, sarcopenia appeared to be an independent predictor of worse survival in selected patients with hepatocellular, pancreatic, and colorectal carcinoma [10–12].

Nevertheless, body composition has received only minor attention in risk analysis for esophageal cancer resection thus far. The aim of the present study is to investigate whether sarcopenia is of prognostic value with regard to postoperative short-term outcome (morbidity and mortality) and long-term survival in patients undergoing esophagectomy for cancer after neoadjuvant chemoradiotherapy (nCRT).

## Materials and methods

### Patient selection and study design

For this study, all patients that took part in the CROSS-I and CROSS-II trials from 2001 to 2012, as well as patients treated accordingly after these trials (post-CROSS cohort), were selected from the institutional database of the Erasmus MC. The CROSS-I trial was a single-center non-randomized phase-2 study conducted in the Erasmus MC and tested the combination of nCRT plus surgery [13]. The CROSS-II multicenter phase-3 trial randomly assigned patients to neoadjuvant CRT followed by esophagectomy, or to surgery alone [14].

Only patients with a CT scan performed not more than 3 months prior to the initial diagnosis of esophageal cancer, but prior to the start of nCRT, and in which the transverse circumference of the body at the level of the third lumbar vertebra was fully visible, were included. In case of multiple relevant scans prior to the start of nCRT, the most recent scan was used.

The Medical Ethical Committee of the Erasmus MC had approved the design of this retrospective study prior to the start of the data analyses. In the current cohort study, the principles of the STROBE statement (Strengthening the Reporting of Observational studies in Epidemiology) have been applied wherever possible.

### Treatments

All patients underwent nCRT, with administration of carboplatin and paclitaxel with concurrent radiotherapy for five weeks [13, 14]. A slight majority of patients underwent a transthoracic esophagectomy (58 %). Posterolateral thoracotomy was the first step in transthoracic resection with extended lymphadenectomy in the chest. During the transhiatal procedure (38 % of patients), the primary tumor and its adjacent lymph nodes were dissected under direct vision through the widened hiatus of the diaphragm up to the level of the inferior pulmonary vein. In addition, all adjacent fatty tissue surrounding the tumor was removed simultaneously, until the lateral resection margins were reached (diaphragm, pleura, pericardium, aorta). After mobilization and transection of the cervical esophagus, the normal intrathoracic esophagus proximal to the primary tumor was mobilized bluntly from the neck to the abdomen with a vein stripper. A gastric tube was created and esophagogastrostomy (hand-sewn or by using a circular stapler) was performed in the neck. The cervical phase of the transthoracic procedure was identical to that of the transhiatal procedure. A minority of patients (3 %) underwent an esophagectomy via a left-sided thoracoabdominal approach. Tumors were assigned pathologic tumor-node-metastasis (TNM) stages according to the Union Internationale Contre le Cancer (UICC) 2002 system (Sixth edition).

### Body composition assessment and analysis

**Fig. 1:**
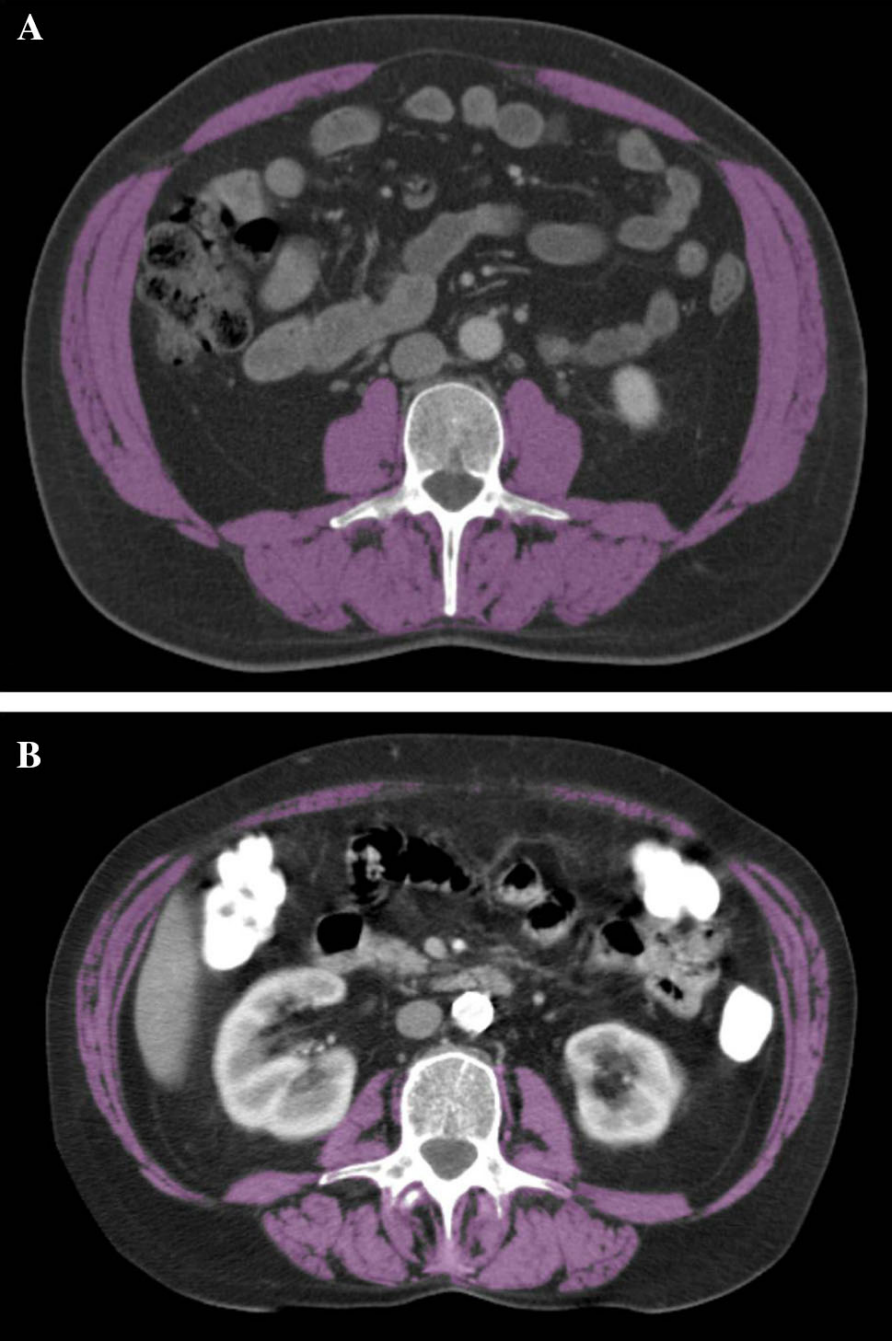
**a, b**. The total cross-sectional transverse area of skeletal muscles was assessed at the caudal end of the third lumbar vertebra (*highlighted in purple*) on a CT scan of both a non-sarcopenic (**a**) and a sarcopenic patient (**b**)

CT scans were used to assess the total cross-sectional transverse areas (cm^2^) of skeletal muscles and visceral adipose tissues at the caudal end of the third lumbar vertebra. Cross-sectional measurements of these tissues at the third lumbar vertebra have been proven to be a good representation of the total body composition[15]. Figure 1 shows an example of this cross-sectional measurement in both a non-sarcopenic (Fig. 1a) and a sarcopenic patient (Fig. 1b).

Images were analyzed using MeVisLab manual segmentation analysis software (Version 4, MeVis Medical Solutions AG, Bremen, Germany). The following Hounsfield Units (HU) were used to accurately distinguish between the different tissues, after manual demarcation of the general desired area of analysis: −30 to +150 HU for skeletal muscle tissue, and −190 to −30 HU for both subcutaneous- and intramuscular adipose tissue [16]. Manual corrections were applied in cases where fibrous tissue would otherwise be considered as skeletal muscle tissue, or where intra-colonic content would be considered as adipose tissue. At the level of the third lumbar vertebra, the following skeletal muscles can be identified: rectus abdominis, external- and internal oblique, transversus abdominis, psoas major, quadratus lumborum, and erector spinae (consisting of the iliocostalis and the longissimus muscles). The classic definition of sarcopenia encompasses a muscle mass which is two standard deviations below that typical of a healthy person. Prado et al. have specifically defined sarcopenia when analyzing patients at the level of the third lumbar vertebra using CT imaging: sarcopenia is present if the total cross-sectional muscle tissue measured transversely at the third lumbar level is less than 52.4 cm^2^/m^2^ body surface area for men and less than 38.5 cm^2^/m^2^ body surface area for women [15]. These definitions were applied to determine whether a patient was sarcopenic or not.

### Statistical analysis

Data were expressed as median values with the range parenthesized. A binary scoring system was used to categorically allocate patients based on the presence or absence of sarcopenia, using the aforementioned reference values for both sexes. Postoperative complications were graded I to V based on severity according to the Dindo–Clavien classification of surgical complications [17] and were considered categorical data. Differences in outcomes between these groups were evaluated using non-parametric tests. Total cross-sectional areas of skeletal muscle and visceral adipose tissues, and their correlation with short- and long-term outcome were analyzed. Overall survival and disease-free survival were depicted through the Kaplan–Meier method, in which the relevant groups were compared using the log-rank test. Association between pretreatment factors and overall survival was determined using univariable Cox regression modeling. Two-sided *p* values ≤0.05 were accepted as statistically significant. Data were analyzed using SPSS statistical analysis software (SPSS Version 19.0, SPSS Inc, Chicago, IL, USA).

## Results

All patients who underwent neoadjuvant treatment according to the CROSS regimen from 2001 to 2012 at the Erasmus MC (*N* = 199) were included in the current study (CROSS-I, CROSS-II, and post-CROSS cohort). In 120 of these patients, an adequate CT scan was available including the complete third lumbar vertebra; all 120 patients received the complete neoadjuvant chemoradiotherapy regimen according to CROSS.

Clinicopathological characteristics are described in Table 1, including surgical data. The 79 excluded patients did not differ in baseline characteristics from the 120 included patients for whom an adequate CT was available (data not shown). Median (range) BMI was 26 kg/m^2^ (15–43 kg/m^2^). Some 54 patients were classified as having sarcopenia (45 %). Of these 54 sarcopenic patients, one patient (2 %) had underweight, 24 patients (44 %) had a normal weight, 24 patients (44 %) had overweight, and five patients (9 %) were obese. Sarcopenic patients had a lower BMI as compared to the non-sarcopenic group: 25 kg/m^2^ versus 28 kg/m^2^, respectively (*p* = 0.001), and were older (64 vs. 61 years, *p* = 0.01). Other clinicopathological characteristics sorted by parameter sarcopenia are displayed in Table 1.

**Table 1:** Clinicopathological characteristics of 120 patients who underwent surgical resection for esophageal cancer after neoadjuvant chemoradiation

	Total *N* = 120	Sarcopenia *N* = 54	No sarcopenia *N* = 66	*p* value
Age^a^ (years)	62 (19–78)	64 (40–78)	59 (19–78)	0.01
Gender				
Male	88 (73 %)	42 (78 %)	46 (70 %)	0.32
Female	32 (27 %)	12 (22 %)	20 (30 %)	
ASA classification				
I	85 (71 %)	44 (82 %)	41 (62 %)	0.02
II	35 (29 %)	10 (18 %)	25 (38 %)	
Operation type				
THE	46 (38 %)	19 (35 %) 32 (59 %) 3 (6 %)	27 (41 %) 38 (57 %) 1 (2 %)	0.31
TPL	4 (3 %)			
TTE	70 (58 %)			
Operation time^a^ (h)	6.5 (3–12)	7.0 (4–12)	6.6 (3–11)	0.30
Histology				
Squamous cell carc.	31 (26 %)	16 (30 %)	15 (23 %) 51 (77 %)	0.39
Adenocarcinoma	89 (74 %)	38 (70 %)		
Radicalism of resection				
R0	110 (92 %)	53 (98 %) 1 (2 %)	57 (86 %) 9 (14 %)	0.02
R1	10 (8 %)			
Pathological ypT-category				
T0	38 (32 %)	18 (33 %) 8 (15 %) 9 (17 %) 18 (33 %) 1 (2 %)	20 (30 %) 9 (14 %) 13 (20 %) 24 (36 %) 0	0.82
T1	17 (14 %)			
T2	22 (18 %)			
T3	42 (35 %)			
T4	1 (1 %)			
Pathological ypN-category				
N0	79 (66 %)	38 (70 %) 15 (28 %) 1 (2 %) 0	41 (62 %) 19 (29 %) 3 (5 %) 3 (5 %)	0.33
N1	34 (28 %)			
N2	4 (3 %)			
N3	3 (3 %)			
Number of resected lymph nodes^a^	17 (4–41)	19 (5–41)	18 (4–39)	0.36
Tumor regression grade^b^				
Major Minor	69 (58 %)	33 (61 %) 21 (39 %)	36 (55 %) 30 (45 %)	0.70
	51 (43 %)			

*ASA classification* American Society of anesthesiologists classification, *THE* transhiatal esophagectomy*, TPL* esophagectomy via left-sided thoracophrenolaparotomy*, TTE* transthoracic esophagectomy

^a^Value presented as median, with its range within brackets

^b^Tumor regression grade according to the Mandard score: major (Mandard 1–2) or minor (Mandard 3–4) regression

Median (range) length of hospital stay was 14 days (7–169). Overall morbidity and in-hospital mortality rates were 73 and 5 %, respectively. Short-term outcome in both sarcopenic and non-sarcopenic patients is shown in Table 2. Overall morbidity rate did not differ between sarcopenic (78 %) and non-sarcopenic (68 %) patients (*p* = 0.24); also in-hospital mortality was not different between the two groups: sarcopenia 6 % versus non-sarcopenia 5 %, *p* = 0.80. The type of complications (*e.g.*, pulmonary or cardiac complications and infections or anastomotic leakage) did also not differ between the groups (data not shown). Furthermore, the histopathological classification of the resection specimen was not different between both groups, although the radicality of the resection was significantly more favorable in the sarcopenic patients (R0-resection =98 vs. 87 % in non-sarcopenic patients, *p* = 0.02).

**Table 2:** Short-term outcome after esophagectomy for cancer; complications were graded according to the Dindo–Clavien classification[17]

	Sarcopenia (*N* = 54)	No sarcopenia (*N* = 66)	*p* value
Overall morbidity	42 (78 %)	45 (68 %)	0.24
Dindo-Clavien			
Grade I	14 (26 %)	10 (15 %)	0.14
Grade II	12 (22 %)	20 (30 %)	0.32
Grade IIIa/IIIb	7/2 (17 %)	3/5 (12 %)	0.48
Grade IVa/IVb	4/0 (7 %)	3/1 (6 %)	0.77
Grade V (mortality)	3 (6 %)	3 (5 %)	0.80
Minor complications (Grade I–IIIB)	35 (65 %)	38 (58 %)	0.42
Major complications (Grade IVa–V)	7 (13 %)	7 (11 %)	0.69
Median hospital stay	14 days (9–169)	14 days (7–115)	0.65

**Fig. 2:**
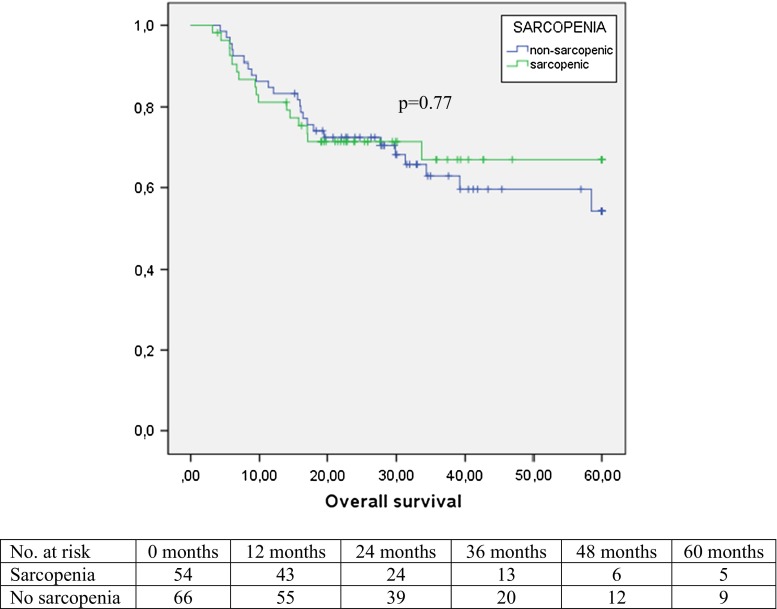
Overall five-year survival in relation to sarcopenia in 120 patients who underwent surgical resection for esophageal cancer after neoadjuvant chemoradiotherapy

Median (range) follow-up in the current patient group was 20 months (0–104). Estimated overall five-year survival was 58 %, whereas disease-specific five-year survival was 66 %. Disease recurrence was noted in 35 patients (29 %): the majority of them (32 patients, 91 %) developed distant metastases. There was no significant difference in overall survival between the patients with or without sarcopenia (*p* = 0.77, Fig.  2) or in disease-free survival (*p* = 0.69).

We compared patients with a low/normal BMI versus the overweight/obese patients in combination with the absence or presence of sarcopenia, in an attempt to identify a subgroup with better or worse outcome after esophagectomy. Results are displayed in Table 3: no increased morbidity or mortality could be identified in sarcopenic patients with overweight or obesity. Finally, we performed univariable Cox regression analysis of pretreatment factors associated with overall survival (Table 4). None of the tested pretreatment factors, including sarcopenia and BMI, were significantly associated with survival in this cohort.

**Table 3:** Short- and long-term outcome of patients according to BMI and sarcopenia

	Sarcopenia (*N* = 54)					No sarcopenia (*N* = 66)				
	*N*	MORB	MORT	OS	DFS	*N*	MORB	MORT	OS	DFS
BMI ≤25 (*N* = 45)	25	56 %	12 %	22 m	22 m	20	60 %	0 %	32 m	29 m
BMI >25 (*N* = 75)	29	55 %	7 %	25 m	23 m	46	54 %	4 %	28 m	26 m
*p* value	NA	0.82	0.52	0.36	0.35	NA	0.67	0.34	0.39	0.41

*N* number of patients in the specific subgroup, *MORB* overall morbidity, *MORT* in-hospital mortality, *OS* median overall survival in months, *DFS* median disease-free survival in months, *NA* not applicable

**Table 4:** Univariable analysis of pretreatment factors associated with overall survival in esophageal cancer patients who underwent neoadjuvant chemoradiotherapy and surgical resection

Pretreatment factors	Univariable analysis		
	HR	95 % CI	*p* value
Age (per decade)	1.09	(0.79–1.52)	0.60
Gender (male vs. female)	1.49	(0.71–3.13)	0.30
ASA classification (I vs. II)	0.82	(0.42–1.59)	0.56
Histology (SCC vs AC)	1.18	(0.59–2.37)	0.63
Clinical N-stage (cN1 vs. cN0)	1.68	(0.82–3.45)	0.15
Sarcopenia (yes vs. no)	0.91	(0.48–1.71)	0.77
BMI (per point)	1.05	(0.98–1.13)	0.15

*HR with 95* *% CI* hazard ratio with 95 % confidence interval, *ASA classification* American Society of Anesthesiologists classification, *SCC* squamous cell carcinoma, *AC* adenocarcinoma

## Discussion

Recently, the impact of specific body compartments (such as skeletal muscle mass) and their prognostic value in the pretreatment phase on postoperative complications and survival has gained interest, mainly due to its modifiable feature in order to potentially improve short- and long-term postoperative outcome. Sarcopenia can be assessed relatively easily on a routine CT scan with no additional patient burden or costs. Also, sarcopenia can be defined by a precise quantification of skeletal muscle mass.

The present study shows that overall morbidity, mortality, and long-term survival are similar between sarcopenic and non-sarcopenic patients in our cohort. This is in line with two previously published studies investigating the role of sarcopenia in small groups of esophageal cancer patients that underwent neoadjuvant chemotherapy [18, 19]. Another study by Sheetz et al. investigated the role of decreased core muscle size in 166 patients that underwent nCRT prior to esophagectomy: no significant association with complications or survival was found [20]. Although the present study could not demonstrate a relationship between sarcopenic obesity and patients’ outcome in the present patient cohort, it might represent a clinically important subgroup of increased risk for worse outcome. The combination of obesity and low muscle mass may influence functional status, chemotherapeutic toxicity, and survival [15]. It may be interesting to perform larger studies to investigate this specific, potentially dismal subgroup of patients in more detail.

Several limitations apply to this study. The current cohort of patients represents a highly selected patient group, of which the majority participated in a clinical trial testing the value of nCRT. Together with the exclusion of patients without adequate CT scans, this undoubtedly has resulted in a selection bias. Also, the more “frail” patient will have been selected not to have surgery; thus, the group selected for surgery (i.e., the population used in the current study) will be fitter compared with total population of esophageal cancer patients in the pretreatment phase. Furthermore, the CT scan, on which the presence or absence of sarcopenia was based, was made prior to the start of nCRT; the influence of the neoadjuvant regimen on the core muscle mass therefore could not be studied. It is possible that changes in core muscle mass directly attributable to nCRT may have confounded the present data. In this light, one can also comment on the interval between the CT scan pretreatment and surgery, which is approximately 4 months. It can be hypothesized that short-term outcome in the postoperative phase may better be predicted by means of a more recent scan just prior to surgery. Finally, sarcopenia is only one of the major features of the frailty syndrome, but does not fully cover it. The frailty phenotype can be defined by the presence of several components besides sarcopenia, such as low physical activity, poor endurance, and weakness, that have not been studied currently. Therefore, further exploration of the frailty syndrome may be of interest in order to identify the potentially modifiable risk factors during the preoperative phase.

In conclusion, the presence of sarcopenia was not associated with a negative short- and long-term outcome in this selected group of esophageal cancer patients after neoadjuvant chemoradiotherapy followed by esophagectomy.
